# The N400 component reflecting semantic and repetition priming of visual scenes is suppressed during the attentional blink

**DOI:** 10.3758/s13414-024-02997-1

**Published:** 2024-12-24

**Authors:** Courtney Guida, Minwoo J. B. Kim, Olivia A. Stibolt, Alyssa Lompado, James E. Hoffman

**Affiliations:** 1 Department of Psychological and Brain Sciences, Boston University, Boston, MA USA; 2 Department of Biomedical Engineering, Ulsan National Institute of Science and Technology, Ulsan, Republic of Korea; 3 Department of Psychology, Florida International University, Miami, FL USA; 4 Department of Psychological and Brain Sciences, University of Delaware, Newark, DE 19716-2577 USA

**Keywords:** Attentional blink, Semantic priming, N400, Automaticity, Conscious awareness

## Abstract

In the attentional blink paradigm, participants attempt to identify two targets appearing in a rapidly presented stream of distractors. Report accuracy is typically high for the first target (T1) while identification of the second target (T2) is impaired when it follows within about 200–400 ms of T1. An important question is whether T2 is processed to a semantic level even when participants are unaware of its identity. We examined this issue in three studies that used natural scenes as stimuli and the N400 component of the event-related potential (ERP) as a measure of semantic priming. In the first experiment, the prime (e.g., a doghouse in a yard) was presented at the beginning of the trial and a test picture that was related (e.g., a dog standing in the kitchen) or unrelated (e.g., a coffee mug on a table) appeared as T2. In the second experiment, the prime was presented as T2 and the test picture appeared at the end of the picture sequence. In both experiments, we found robust semantic priming when participants were aware of the identity of the blinked picture and an absence of priming when they were unaware. In Experiment 3, we used identity priming to assess whether earlier representations preceding semantics were preserved, and again found that priming critically depended on awareness of the prime’s identity. These results suggest that semantic priming in scenes, as measured with the N400, is a higher-level process that critically depends on attention and awareness.

## Introduction

An important question in attention research concerns the role that conscious awareness plays in the processing of visual stimuli. According to two-stage theories of visual processing (Chun & Potter, 1995; Hoffman, 1978, 1979; Wolfe et al., 1989), the initial stage in visual processing is parallel and unlimited in capacity, allowing all objects to receive low-level analysis, perhaps consisting of simple feature extraction such as shape and color (see Wolfe & Horowitz, 2004, for a list of potential features extracted in early vision). Features analyzed in the first stage can be used to guide attention allocation to potentially important objects that pass through a “bottleneck” to a second, capacity-limited stage that plays an important role in conscious awareness, working memory, and other higher-level processes. Much of the debate in early versus late theories of attention was concerned with the nature of representations in these two stages. For example, does identification of an object (this bundle of features is an “apple”) occur in stage 1 processing, or does it require higher-level processes in stage 2? Similarly, we can ask which stage is responsible for various cognitive processes such as deriving the meaning of visual objects, consolidating information into working memory, and even awareness itself (e.g., Dembski et al., 2021).

### The attentional blink

These issues are central to understanding phenomena such as the attentional blink (AB), which was initially discovered by Broadbent and Broadbent (1987) and explored subsequently by Shapiro and colleagues (1997b; see Dux & Marois, 2009, and Martens & Wyble, 2010, for reviews). The AB occurs when individuals search for two target stimuli embedded in a rapid, serial, visual presentation (RSVP) stream of distractors. Although participants are typically highly accurate in identifying the first target (T1), the second target (T2) is often missed if it follows within approximately 200–400 ms of the first. Several theories suggest that the AB reflects the bottleneck in visual processing where the unlimited, parallel processing of features in stage 1 interfaces with the limited-capacity stage 2 processing that is important for memory consolidation, response selection, and perhaps conscious awareness. In this model, the blink occurs because the bottleneck is still occupied with the first target when the second one arrives. The second target then must wait for access to the bottleneck and is vulnerable to masking and replacement by subsequent stimuli in the stream (i.e., it has been “blinked”). Following the discovery of the AB, a variety of alternative theories have been proposed (see Di Lollo et al., 2005; Olivers et al., 2007; Taatgen et al., 2009; Wyble et al., 2009), but this paper continues to use the bottleneck model to guide an exploration of several important issues raised in the AB literature. For example, a key question concerns the nature of the representations achieved by blinked items in the initial parallel stage. Are blinked objects represented solely in terms of low-level features such as shape and color, or is information about identity and meaning included?

Shapiro et al., (1997a, 1997b) used semantic priming to address this question. The task consisted of a modified AB paradigm in which participants reported three consecutive target words, each separated by two distractor words; importantly, the second target (T2) could be semantically related or unrelated to T3. The question was whether a T2 word that was blinked by a preceding T1 would still exert semantic priming on T3. The answer was affirmative, with higher accuracy in reporting T3 words that were semantically related to T2 compared to unrelated T3 words, even when T2 was not correctly reported. This result is consistent with several other studies showing that masked words that could not be reported still served as semantic primes for following words (Allport, 1977; Forster & Davis, 1984; Marcel, 1983). Similar results have been reported for subliminally presented pictures (Carr et al., 1982; Dell’Acqua & Grainger, 1999; McCauley et al., 1980; van den Bussche et al., 2009). Unfortunately, these various subliminal semantic priming effects do not always replicate (e.g., Kang et al., 2011; Stein et al., 2020; Yang et al., 2017). We return to the issue of replicability in the *Discussion*.

An alternative approach to determining the depth of processing of blinked items in the AB is to examine measures of brain activity such as event-related potentials (ERPs). These measures differ in their latency and neural origin, which can help determine whether the blink is affecting perceptual processing or higher-level stages associated with working memory and awareness (Vogel et al., 1998; Zivony & Lamy, 2022). For example, the P1 component of the ERP appears approximately 80–100 ms after presentation of a visual stimulus and is generated in the extrastriate visual cortex (Hillyard & Picton, 1987). If the amplitude of the P1 component elicited by T2 was reduced during the blink, it would support the claim that the AB can suppress representations of stimuli in early, perceptual processing stages. This does not appear to be the case, as the amplitude of the P1 generated by T2 is unaffected by the AB (see Zivony & Lamy, 2022, for a review).

The issue of whether *semantic processing* is suppressed during the blink can be examined using the N400 component of the ERP (Batterink et al., 2010; Giesbrecht et al., 2007; Lien et al., 2008; Nigam et al., 1992; Rolke et al., 2001; Vachon & Jolicœur, 2011; Vogel et al., 1998). For example, Experiment 2 in Vogel et al., (1998; see also Luck et al., 1996) had participants view a prime word presented at the beginning of each trial (e.g., “SHOE”) followed by a rapidly presented stream of blue, random letter strings appearing at fixation. Each stream contained a T1 consisting of a string of digits (e.g., “7,777,777”) followed by a red T2 word that was related (“SOCK”) or unrelated (“DESK”) to the prime. The task was to report whether T1 was even or odd and whether T2 was related or unrelated to the prime. They found that T2 accuracy was high when it appeared immediately following T1 (a phenomenon known as lag 1 sparing) and when there were seven intervening words (lag 8). At the intermediate lag (two intervening words, or lag 3), performance was severely impaired, reflecting the AB. These behavioral results are similar to findings obtained in previous AB studies with words (Broadbent & Broadbent, 1987; Luck et al., 1996; Maki et al., 1997). Surprisingly, however, the amplitude of the N400 was unaffected by lag, indicating that semantic priming was independent of conscious awareness (as reflected in report accuracy) of the T2 word. This remarkable result provides strong support for the claim that meaning may be extracted in the early, parallel, and automatic stage of processing rather than in the late, limited-capacity central stage responsible for awareness and working memory. In the *Discussion*, we review subsequent attempts to replicate this result.

### Automaticity of scene recognition

In the present work, we used a paradigm similar in several respects to that of Experiment 2 in Vogel et al. (1998) to determine whether the meaning of scenes can also be extracted during Stage 1 processing. Scene recognition and categorization is likely the most practiced visual identification process of all, as we view visual scenes virtually every waking second. This extensive experience could potentially result in this process becoming automatic, allowing it to occur without attention and/or awareness (Moors, 2016; Schneider & Shiffrin, 1977). This would make recognizing and understanding the gist of scenes a potential candidate for automatic processing in the absence of attention and awareness. On the other hand, visual scenes are complicated, consisting of multiple objects with occlusion and varying viewpoints which might require attention and higher-level processing.

Research on the automaticity of scene processing has yielded mixed results. Consistent with our extensive experience with visual scene recognition, it is remarkably rapid. For example, in two conditions, Potter (1975) had participants view an RSVP sequence of scenes presented at a rate of 125 ms per picture. In the search condition, they had to monitor the sequence for a particular target (e.g., a boat), while in the memory condition, they were given the target name *after* the sequence was presented. Targets were defined by providing participants with a preview of the target scene or by giving them a categorical verbal description such as “a boat.” Note that verbal descriptions were used in order to eliminate search based on low-level physical features. Visual search for a known scene was quite good (70%) regardless of whether the target was designated by its name or by a preview of the actual scene, suggesting that observers were activating a conceptual representation of each picture, despite the short presentation durations. In contrast, *memory* for pictures at this rate was quite poor (10%), showing that memory consolidation is a highly capacity-limited process that can only be applied to a few of the pictures in the sequence.

Taken together, good performance in the search task with a named target coupled with poor performance in the memory tasks suggests that pictures were briefly understood but rapidly forgotten. In an effort to further eliminate the use of low-level features in search, Intraub (1981) employed a “negative” category search task (e.g., “detect the picture that is not of food”). With a presentation rate of 114 ms per picture, participants were still able to detect 35% of the targets, consistent with the claim that at least some of the pictures were processed to a conceptual level. Conceptual processing of pictures may occur with even shorter exposure durations. Potter et al. (2014) had participants search RSVP picture sequences for targets that were defined in terms of a short verbal prompt (e.g., “smiling couple”), and found above-chance performance with durations as short as 27 ms. However, Maguire and Howe (2016) found that this result depended on using distractor pictures in the sequence that contained considerable “white space,” perhaps allowing for the critical picture to persist in iconic memory beyond its short exposure duration. Nonetheless, they found that using pictures without blank areas resulted in above-chance detection of picture meaning at durations of 40–50 ms, which is still remarkably fast.

Thorpe and colleagues (1996) showed that superordinate level objects such as “animal” could be detected in natural scenes with durations as short as 20–25 ms. They also reported that this discrimination was associated with an ERP component with an onset of approximately 150 ms, providing a lower bound on the onset time of brain processes required to perform this categorization. Remarkably, this rapid classification can be accomplished as quickly for two simultaneously presented scenes as for one (Rousselet et al., 2004), suggesting that capacity limits on scene categorization may exceed a single picture.

Although these results attest to the remarkable speed at which pictures are comprehended, they do not address the role of attention in scene recognition. This issue has been directly investigated in several dual-task studies. For example, Li et al. (2002) had participants perform a difficult visual search task (detect an “L” in a briefly presented display of “T’s”) while simultaneously determining whether a briefly presented scene contained an animal. They found that dual-task performance was not significantly different from performance on each of the tasks when they were performed alone. In contrast, there was severe dual-task interference when the scene task was replaced by a simple perceptual task that is known to require visual attention (determining the spatial arrangement of colored halves of a disk), leading Li et al. to conclude that scene categorization can be accomplished with little or no attention. However, this may be something of a special case. Walker et al. (2008) confirmed that only minimal attention was required for people to detect an animal in a scene, but only when the animal was the only foreground object. Multiple foreground objects yielded considerably larger impairments in the “near absence of attention.”

Similarly, Cohen et al. (2011) asked participants to track a set of moving objects while attempting to detect an animal or a vehicle appearing in a background scene. Performance in the detection task was only impaired when the tracking task was difficult. These various studies strongly suggest that *conscious perception* of a scene depends on attention. However, the question of whether *unconscious semantic processing* of scenes may occur automatically without attention remains open. The present series of studies were designed to address this issue.

## Experiment 1

**Fig. 1:**
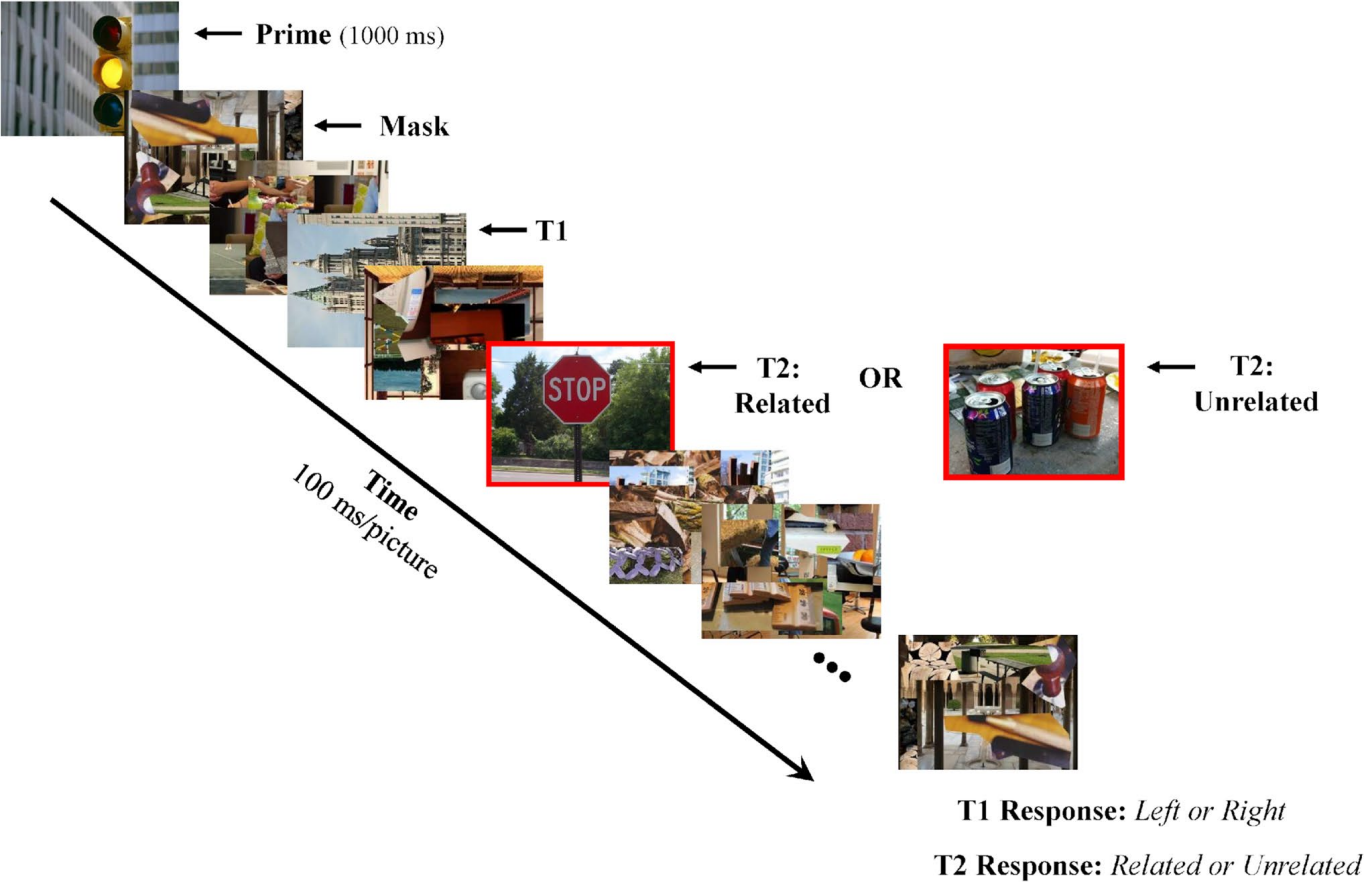
A typical trial in Experiment 1. A prime picture was shown for 1,000 ms prior to the rapid, serial, visual presentation (RSVP) stream, which consisted of two target pictures embedded in a series of pictures composed of scrambled scene fragments (“masks”). The first target (T1) was a landscape or cityscape image rotated 90° to the left or right. The second target (T2) was a scene picture (cued by a red rectangle) that was semantically related or unrelated to the prime picture. T2 appeared two or eight pictures (lag 2 or lag 8) after T1. Following the sequence, participants indicated the orientation of T1 followed by the relatedness of T2 and their confidence in their T2 response

The current study was partly based on the paradigm used in Luck et al. (1996) and Experiment 2 of Vogel et al. (1998), but employed natural scene stimuli in place of linguistic materials (see Fig. 1 for the procedure). The trial started with a prime picture shown for a duration of 1,000 ms followed by a sequence of pictures containing filler pictures (scrambled scene pictures), a “T1” picture, and a “T2” picture that was related or unrelated to the prime. The T1 task was to determine whether a scene picture was rotated 90° to the left or right. The T2 task was to determine whether the cued picture was semantically related or unrelated to the prime picture presented at the beginning of the trial. To reduce errors associated with failures to attend to the correct picture in the sequence, T2 was cued by a red rectangle and was the only coherent scene picture besides T1 that appeared in the stream. The presence of an AB was assessed by examining the effect of lag on T2 accuracy while the amplitude of the N400 elicited by T2 provided a measure of semantic priming. It should be noted that accuracy in judging the semantic relatedness of the prime and test pictures was collapsed over related and unrelated trials, a procedure used in other studies that employed this task (e.g., Batterink et al., 2010; Giesbrecht, et al., 2007; Kang et al., 2011; Luck et al., 1996; Vachon & Jolicœur, 2011; Vogel et al., 1998, Experiment 2).

### Methods

#### Participants

Although Vogel et al. (1998) did not report effect sizes due to the convention at the time, a later AB study from Vachon and Jolicœur (2011) reported a main effect of lag on the N400 amplitude with an effect size of 0.518 partial eta squared (converted from 1.01 Cohen’s *d*). G*power (Erdfelder et al., 1996) revealed that at least 15 participants were required for detecting a minimum effect size of 0.518 partial eta squared with 0.80 power and 0.05 alpha for three conditions (in our case, the conditions are lag 8 correct, lag 2 correct, and lag 2 incorrect). Each of the three experiments in the current paper used 20 participants, which is more than the required sample size for detecting lag-related modulation of the N400. In addition, 20 participants were equivalent to the number of participants in Vachon and Jolicœur (2011) and more than Vogel et al. (1998), who used 14 participants in their N400 experiment (Experiment 2). Sample size was further consistent with previous work in our lab using similar paradigms, such as ERPs in emotion-induced blindness (e.g., Hoffman et al., 2020; Kennedy et al., 2014).

Participants were recruited from the University of Delaware and surrounding community, and they were compensated $10 an hour for their time. The University of Delaware Institutional Review Board approved the study (Protocol #921,456–5), and all participants provided informed consent. Twenty individuals participated in the study (ages 19–27 years; nine males, 11 females). All participants reported normal or corrected-to-normal acuity with no history of neurological disorders. For a subject to be included in the final data set, 70% of their total EEG segments had to be usable. On average 60.75 (9.49%) epochs were removed due to either ocular artifacts or excessive noise in the EEG data (see Table 1 for information on the number of trials used to compute ERPs in each condition for all three experiments). None of the data or materials for the experiments reported here are available online, but they can be provided upon request. The experiment was not pre-registered.

**Table 1:** Number of trials in each condition per experiment

	Condition	M	SD	Range
Experiment 1				
	Lag 2 incorrect	97.3	33.7	45–160
	Lag 8 incorrect	43.2	17.87	19–86
	Lag 2 correct	162.9	36.4	65–212
	Lag 8 correct	215.4	29.5	160–263
Experiment 2				
	Lag 2 incorrect	66.7	18.16	42–107
	Lag 8 incorrect	98.9	18.86	55–128
	Lag 2 correct	213.45	24.78	178–255
	Lag 8 correct	165.4	22.97	123–226
Experiment 3				
	Lag 2 incorrect	88.4	32.75	50–197
	Lag 8 incorrect	36.65	19.71	14–91
	Lag 2 correct	189.4	33.12	80–232
	Lag 8 correct	227.45	23.33	182–276

Number of trials per condition after removing trials with incorrect T1 and EEG/ocular artifacts.

#### Stimuli

The main stimulus set was composed of 640 sets of three pictures each. We used an additional 16 sets for practice. Each set consisted of a prime picture and two test pictures that were semantically related/unrelated to the prime picture. Each picture in the set contained a central object that was the basis for the relatedness judgment. For the majority of the prime and test pictures, the objects were loosely based on the related and unrelated object pictures found in the Bank of Standardized Stimuli (BOSS; Brodeur et al., 2010), except we used scenes obtained from the internet that contained these objects. To create additional pairings, we used standardized lists of related words (e.g., Merriam-Webster Thesaurus, 2006) and found scenes on the internet containing the corresponding objects.

There were 20 filler or mask pictures that were created manually by cutting out six to ten rectangular fragments varying randomly in size from six scene images that were reserved for this purpose. These fragments were then randomly rotated 0°, 90°, 180°, or 225° and pasted in random locations on one of the six original images. These mask images were no longer coherent scene pictures but were similar enough to the target pictures to make the task challenging.

We collected subjective relatedness ratings of our stimulus set from a separate sample of 40 participants who were tested online. Participants were recruited from the University of Delaware subject pool and received two credit hours toward their participation requirement. Two subjects were excluded due to a failure to follow instructions and they were replaced with two additional participants.

Pairs of related and unrelated images from our stimulus set were displayed side by side in random order while participants judged their semantic similarity using an on-screen slider that could vary between 1 (“unrelated”) and 9 (“very closely related”). Related and unrelated pairs occurred equally often and images were not repeated. A dependent-measures *t*-test revealed that the related pictures were rated significantly higher in semantic relatedness (*M* = 7.90, *SE* = 0.04) than the unrelated pictures (*M* = 1.97, *SE* = 0.04, *t*(297) = 98.52, *p* < 0.001). These ratings provide objective support for the claim that our pictures differed in their semantic relatedness.

#### Design and procedure

In the main experiment, participants were seated in a dimly lit, electrically shielded, and acoustically isolated room. The stimuli were presented on a 1,680 × 1,050-pixel resolution Samsung 2233RZ 22-in. LCD Monitor with a refresh rate of 120 Hz. The experiment was controlled by a Dell 3.60 GHz computer and programmed using Python 2.7 (Python Software Foundation, https://www.python.org/) including PsychoPy2 extension modules (Peirce et al., 2019). A viewing distance of 74 cm from the monitor was maintained across subjects using a chin rest. An SR Eyelink 1000 eye-tracker monitored eye blinks and eye movements, sampling eye position at 500 Hz.

Subjects completed a series of ten RSVP practice trials prior to the beginning of the experiment. The practice trials began with presentation of the prime picture for 1,000 ms followed by an RSVP sequence of images presented at a rate of five images per second. This rate gradually increased to the rate used in the actual experiment, which was ten images per second. The main experiment consisted of 640 trials. Each trial began with a centrally presented red fixation cross that remained visible throughout the duration of the trial. Participants initiate each trial by clicking on the left mouse button. They were instructed to maintain fixation on the cross for the entire trial and avoid blinking or making any unnecessary movements. Eye-tracking data were used to provide participants with feedback at the end of each trial regarding blinks and eye movements, and trials containing these artifacts were not included in the analyses.

Images were colored photographs centered on the fixation cross. Each image was 480 × 360-pixels and subtended 11.4 × 8.6 visual degree angle (vda). The RSVP stream consisted of a sequence of 13–24 pictures presented at a rate of ten pictures per second. T1 was a picture of a landscape or cityscape rotated 90° to the left or right that appeared at stream positions 2, 3, 4, or 5. T2 was a coherent scene surrounded by a red rectangle and was related or unrelated to the prime picture presented at the beginning of the trial. It occurred either two (lag 2) or eight (lag 8) images after T1. T2 was followed by eight filler pictures for both lags to ensure that the time required to hold information about the relatedness of T2 would be constant for the two lags. As noted above, the filler pictures in the stream (“masks”) consisted of scrambled fragments from a set of scenes reserved for this purpose.

Following the picture sequence, participants responded by using the mouse to click on graphical buttons appearing on the screen. The T1 response indicated whether the landscape picture was rotated to the right or left. Participants were instructed to try to maintain 90% accuracy on this task and they were provided with feedback on their T1 performance after each block of 80 trials. Trials in which T1 was incorrectly reported were excluded from all analyses. The T2 response indicated whether the T2 picture was semantically related or unrelated to the prime picture. Following the T2 response, participants rated their confidence in their judgment by clicking buttons labeled *Sure, Not Sure*, or *Guess*. Following the relatedness response, feedback was provided in the form of a green *Correct* or red *Incorrect* label. *Guess* trials were treated as incorrect during the subsequent data analyses.

Participants were randomly assigned to one of two groups when they arrived for the study. For one set of participants, the order of pictures and conditions (related vs. unrelated pair and lag) was random. Each of the remaining participants was paired with a participant in the first set, such that they received the same order of pictures and lags, but the assignment of *related* versus *unrelated* was reversed.

#### Electrophysiological recording and analysis

An Electrical Geodesics Inc. system (EGI; Eugene, OR) Net Amps 200 amplifier was used to record the electroencephalogram (EEG) with 128 channel Hydrocel Sensor Nets and EGI Net Station 4.5.6 software. Following manufacturer recommendations, we maintained individual sensor impedances below 75kΩ. The vertex served as the reference and EEG was band-pass filtered (0.01–80 Hz) online.

Pre-processing and analysis of the data were performed using MATLAB with the EEGLAB toolbox extension. A 0.1- to 40-Hz band-pass filter was applied to reduce movement and noise artifacts from the data. Channels that were marked as bad were removed and replaced using interpolation of surrounding channels. The data were then segmented into epochs beginning 200 ms prior to and ending 1,000 ms after onset of the T2 picture. Bad epochs were rejected using visual inspection as well as the ASR (artifactual substance reconstruction) and TBT EEGLAB plugin (v2.6.0; Ben-Shachar, 2020). Processed segments were then re-referenced to an average reference and grand averaged over trials.

Difference waveforms (*unrelated* – *related*) were computed for correct and incorrect T2 trials at each of the two lags. Difference curves were also computed based on all trials regardless of accuracy. See Table 1 for a summary of trials included in each condition per experiment. An a priori minimum of 20 trials in the lag 2 incorrect condition was required for participants to be included in the analysis, but the actual minimum trial counts exceeded this minimum by a healthy margin. We did not include lag 8 incorrect trials in the analyses in this experiment or the following experiments due to the small number of trials in this condition.

The N400 was defined as the mean amplitude of the difference between *unrelated* and *related* ERPs (*unrelated* – *related* for Experiments 1 and 2 and *different* – *same* for Experiment 3) across a set of time points using an array of sensors centered on Cz including CPz, FC2, FCz, C1, CP1, CP2, C2 in the international 10–10 system (E7, E106, E80, E55, E31, E79, E54, E87, E37, E30, E105, E13, E112, E6 in the EGI system) that were chosen a priori based on earlier N400 studies (see Šoškić et al., 2022). Using the guidelines proposed by Šoškić and colleagues (2022), we chose averaging time windows that ranged from 300 to 500 ms for Experiment 2 and Experiment 3. However, in Experiment 1, the N400s for the lag 2 condition were clearly delayed compared to the lag 8 condition. For this reason, two averaging windows for Experiment 1 were selected a priori based on Vachon and Jolicœur (2011), who observed similar delays in the N400: an *early* window of 450–650 ms and a *late* window of 550–800 ms. An averaging window of 450–650 ms was used in additional analyses performed on the N400 collapsed across correct and incorrect trials to facilitate comparison to Luck et al. (1996) and Experiment 2 of Vogel et al. (1998).

One-way repeated-measures ANOVA and Bonferroni post hoc analyses were used to assess statistical differences between conditions. Single-sample *t*-tests were used to assess the significance of the N400 amplitudes compared to zero microvolts. One-sample Bayesian *t*-tests using the inverse Bayes factor were conducted on N400 amplitude in the lag 2 incorrect condition in order to assess the statistical evidence favoring the null hypothesis that the N400 amplitude on error trials was zero (i.e., an absence of the N400 component on error trials).

### Results

#### Behavioral results

A correct trial was defined by the accurate judgment of T1 orientation together with a correct report of the semantic relatedness of the prime and test (T2) pictures. The relatedness judgment required a confidence judgment of *Sure* or *Not Sure* to be scored as correct. Guesses were scored as incorrect. As expected, T1 report accuracy was high and was similar for the two lags (lag 2: *M* = 87.79%, *SE* = 0.95%; lag 8: *M* = 88.64%, *SE* = 1.06%). The presence of a strong AB was revealed by lower accuracy in judging the semantic relatedness of T2 pictures at lag 2 (*M* = 62.2%, *SE* = 2.9%) compared to lag 8 (*M* = 82.6%, *SE* = 1.7%). This difference was significant as shown by a repeated-measures t-test, *t*(19) = 7.755, *p* < 0.001, *d* = 1.734.

#### Event-related potential (ERP) results

**Fig. 2:**
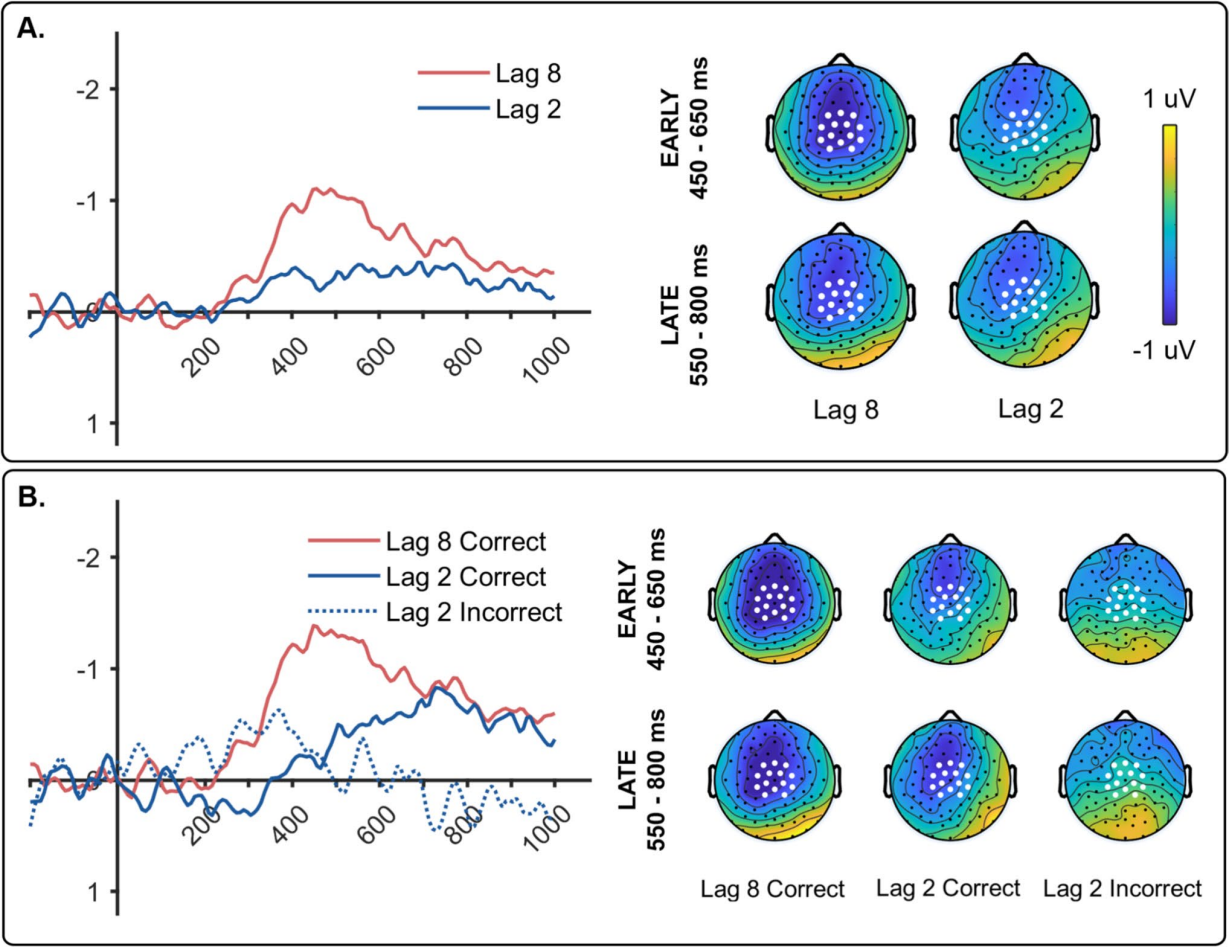
**(A)** The N400 waveform (**left**) and topo map (**right**) for lag 8 and lag 2 trials combined across correct and incorrect. Topo maps are from early (450–650 ms) and late (550–800 ms) measurement windows. The sensors used for measuring mean amplitude are indicated by white dots. **(B)** The N400 waveform (**left**) and topo map (**right**) from Experiment 1 elicited by the test picture (T2) separated according to whether T2 was correct or incorrect

The N400 component generated by T2 was isolated by subtracting related from unrelated ERPs (*unrelated* – *related* subtraction). N400 waveforms for lag 2 and lag 8, collapsed across correct and incorrect trials, are shown in Fig. 2A along with the averaged topographic maps for the N400. A paired *t*-test revealed that the N400 was larger for lag 8 compared to lag 2, *t*(19) = 3.017, *p* = 0.007, *d* = 0.675. Single-sample *t*-tests also showed that the N400s for lags 2 and 8 were both significantly different from zero: lag 2, *t*(19) = 2.710, *p* = 0.014,* d* = 0.606; lag 8, *t*(19) = 5.552, *p* < 0.001, *d* = 1.241.

N400s were also examined separately for correct and incorrect trials and are shown in Fig. 2B (lag 8 incorrect was excluded because of the small number of trials in this condition). The N400 for the lag 2 correct condition is clearly delayed (delay of 150 ms) relative to lag 8 correct (see Vachon & Jolicœur, 2011, for a similar delay of the N400 during the AB). We evaluated this delay using the jackknife procedure (Luck, 2014) to compare the 50% area latencies of the N400 in the lag 2 and lag 8 correct conditions. We found that the N400 latency was longer for lag 2 correct (*M* = 729 ms, *SE* = 2.12 ms) compared to lag 8 correct (*M* = 579 ms, *SE* = 1.579 ms), as revealed by a paired-sample *t*-test (*t*(19) = 5.612, *p* < 0.001, *d* = 1.25).

Consequently, we used two different time windows to analyze N400 amplitude: an early window extending from 450 to 650 ms and a late window extending from 550 to 800 ms. For the early window, a repeated-measures ANOVA revealed a significant effect across conditions (lag 2 correct, lag 2 incorrect, lag 8 correct), *F*(2, 38) = 10.532, *p* < 0.001, η^2^_*p*_ = 0.357. Bonferroni post hoc tests showed that the N400 for lag 8 correct was significantly larger compared to lag 2 correct (*p* = 0.008), and lag 2 incorrect (*p* < 0.001). There was no significant difference between N400 amplitudes for lag 2 correct versus lag 2 incorrect (*p* > 0.05)*.*

There was also a significant effect across conditions in the late time window, *F*(2, 38) = 7.568, *p* = 0.002, η^2^_*p*_ = 0.285. Post hoc tests showed that the N400s for lag 8 correct (*p* = 0.002) and lag 2 correct (*p* = 0.024) were both larger compared to lag 2 incorrect, but did not differ from each other (*p* > 0.05). In order to determine whether correct N400s were the same across the two lags, we compared the lag 8 correct N400 from the earlier time window to the lag 2 correct N400 from the later window, and found the lag 8 N400 to be significantly larger, *t*(19) = 3.836, *p* = 0.001, *d* = 0.858.

A series of single-sample *t*-tests compared N400 amplitude to zero to determine whether the N400 was present or absent in each condition. This revealed significant N400 amplitudes for lag 8 correct in both time windows (Early: *t*(19) = 6.201, *p* < 0.001, *d* = 1.387; Late: *t*(19) = 5.006, *p* < 0.001, *d* = 1.119). The N400 amplitude for lag 2 correct reached significance in both the early window (*t*(19) = 2.129, *p* = 0.047, *d* = 0.476), and the late window (*t*(19) = 3.165, *p* = 0.005, *d* = 0.708). In contrast, the N400 for lag 2 incorrect failed to show significant differences from zero in either time window (early: *t*(19) = 0.716, *p* = 0.482, *d* = 0.160; late: *t*(19) = 0.314, *p* = 0.757, *d* = 0.070).

We followed up the lack of significant N400 amplitude in the lag 2 incorrect condition with a two-tailed Bayesian one-sample *t*-test against zero microvolts. This yielded inverse Bayes factors of 3.422 and 4.116 in early and late windows, respectively, which provides “substantial” evidence in favor of the null hypothesis that semantic priming, as reflected in the N400 component, was eliminated when participants were incorrect in judging the semantic relationship between the prime and test pictures.

### Discussion

In Experiment 1, we used the AB to control the amount of attention allocated to a target scene that had been preceded by a related or unrelated prime picture. We found that the amplitude of the N400 component was significantly modulated by the AB, with a larger N400 in the lag 8 compared to the lag 2 condition. These results are quite different from those reported in Luck et al. (1996) and Experiment 2 of Vogel et al. (1998), who used visually presented words. They found that the amplitude of the N400 amplitude was the same across lags even though the accuracy in *reporting* the relatedness of the prime and test pictures was severely impaired by the AB. In addition, we found that, during the blink, the N400 on incorrect trials was significantly smaller than the N400 on correct trials and was not significantly different from zero, suggesting that the N400 depends on conscious awareness of the identity of the T2 picture (note that throughout this paper we are assuming that correct report of the relatedness of the prime and test scenes depends on awareness of the *identity* of these scenes).

The current results are consistent with various two-stage models of information processing (e.g., Chun & Potter, 1995; Hoffman, 1978, 1979; Jolicœur et al., 2001) in which information extracted in stage 1 must pass through a central bottleneck that limits access to “higher-level processing” in stage 2. If the bottleneck is occupied by an initial target (T1), a closely following second target (T2) has to wait until the bottleneck is free. On some trials, if T2 is masked, it may no longer be able to access the bottleneck once it becomes available. On other trials, it may enter the bottleneck after some delay resulting in an increase in T2 reaction time as well as the latency of T2 ERP components that are generated by processes that follow the bottleneck. The pattern of delay and suppression effects we observed for the N400 in Experiment 1 clearly supports the idea that semantic analysis of T2 reflected in the N400 component occurs in stage 2 rather than in the parallel and automatic processes of stage 1. In this regard, the N400 appears to be similar to the P3b component of the ERP, which is thought to reflect consolidation of information into working memory. Like the N400, the P3b elicited by targets during the AB is suppressed (Vogel et al., 1998) and delayed (Vogel & Luck, 2002), suggesting that awareness, extraction of scene semantics, and working memory consolidation may all occur in stage 2.

## Experiment 2

**Fig. 3:**
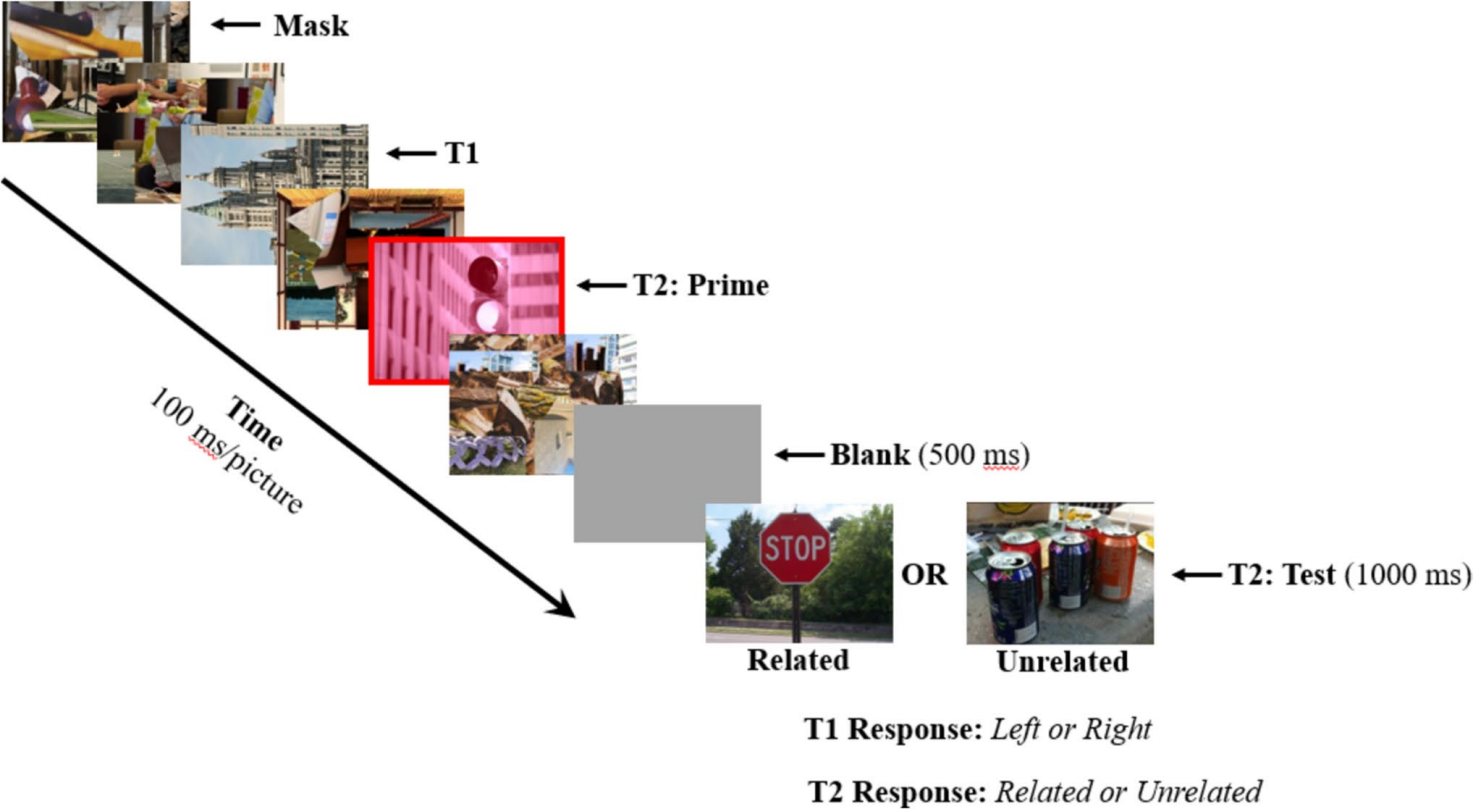
The attentional blink (AB) paradigm used in Experiment 2. The sequence of pictures on each trial consisted of a series of masks and two targets (T1 and T2). T1 was a landscape or cityscape image that was rotated 90° to the left or right. T2 was the prime picture which was cued by a red tint and a surrounding red rectangle. appearing at lag 2 or lag 8. The T2 prime picture was followed by a single mask and then a blank period of 500 ms. The stream ended with a final *test picture* that was either semantically related or unrelated to the T2 prime picture

Experiment 2 was designed to examine the generality of the association between semantic priming and awareness. In this experiment, the *prime* picture is presented at lag 2 and the *test* picture, which is semantically related or unrelated to the prime, is presented for a long duration at the end of the RSVP sequence (see Fig. 3). This procedure has the virtue of avoiding the delay in the N400 when the target picture occurs in close temporal proximity to T1, as was the case in Experiment 1. This should allow a clearer examination of N400 amplitude in the various conditions. Importantly, this method is designed to assess whether awareness is also required to *produce* semantic priming. In Experiment 1, the prime picture was presented at the beginning of the trial for a long duration, ensuring full identification and, presumably, activation of semantically related concepts. The N400 elicited by the T2 picture depended on whether semantically related representations elicited by the earlier prime picture were related to the meaning of T2. In Experiment 2, we examined whether a T2 scene picture that is blinked at a short lag after T1 can still *produce* activation of semantically related concepts. If so, these primed representations should influence the amplitude of the N400 elicited by a related versus unrelated T2 scene appearing at the end of the trial (for a similar procedure, see Peressotti et al., 2012; Pesciarelli et al., 2007; Rolke et al., 2001).

### Methods

#### Participants

Twenty-eight individuals with normal or corrected-to-normal vision and no history of neurological disorder participated in the study (ages 19–28 years; 12 males, 16 females). Six participants were excluded from analysis due to an inability to achieve at least 75% accuracy in reporting T1. An additional two participants were excluded from analysis due to having too few usable segments because of excessive movements or noisy EEG recordings (defined as more than 40% rejected trials). The final sample size consisted of 20 participants (ages 19–28 years; nine males, 11 females). On average, participants had 37.15 (5.80%) of their epochs excluded due to ocular artifacts and excessive noise.

#### Design and procedure

The apparatus, stimuli, and procedures for data collection and analysis were similar to those used in Experiment 1 except for the following. The *prime* picture served as the second target (T2) and was tinted red (see Fig. 3) to ensure that participants had clear information about which picture in the sequence was the T2. In addition, T2 was followed by a single mask picture and then a 500-ms blank screen before the test picture appeared for 1,000 ms. This method is similar to that of Sergent et al. (2005), who used a “reduced AB design” consisting of two targets each followed by a mask. One advantage of this procedure is that it potentially reduces interference from irrelevant scene pictures appearing after T2.

In contrast to Experiment 1, we found that the average latencies of the N400 for correct trials at lags 2 and 8 were not significantly different (*t*(19) = 1.05, *p* > 0.05). Therefore, we adopted a commonly used averaging time window (300–500 ms; Šoškić et al., 2022) for measuring the N400 in Experiment 2. The rest of the recording and analysis procedures for Experiment 2 were the same as Experiment 1.

### Results

#### Behavioral results

A correct trial was defined by the correct discrimination of T1 orientation (lag 2: *M* = 93.77%, *SE* = 0.77%; lag 8: *M* = 94.65%, *SE* = 0.58%) and a correct relatedness judgment of T2. In addition, the confidence of the relatedness judgment had to be *Sure* or *Not Sure* to be considered a correct trial; *Guess* confidence judgments were scored as incorrect. The average percentage accuracy averaged over semantic relatedness of the image pairs was significantly lower at lag 2 (*M* = 62.6%, *SE* = 2.2%) compared to lag 8 (*M* = 85.4%, *SE* = 1.3%), as revealed by a repeated-measures two-sample *t*-test (*t*(19) = 11.822, *p* < 0.001, *d* = 2.643). These results are similar to those of Experiment 1 and confirm the presence of a substantial AB effect.

#### ERP results

**Fig. 4:**
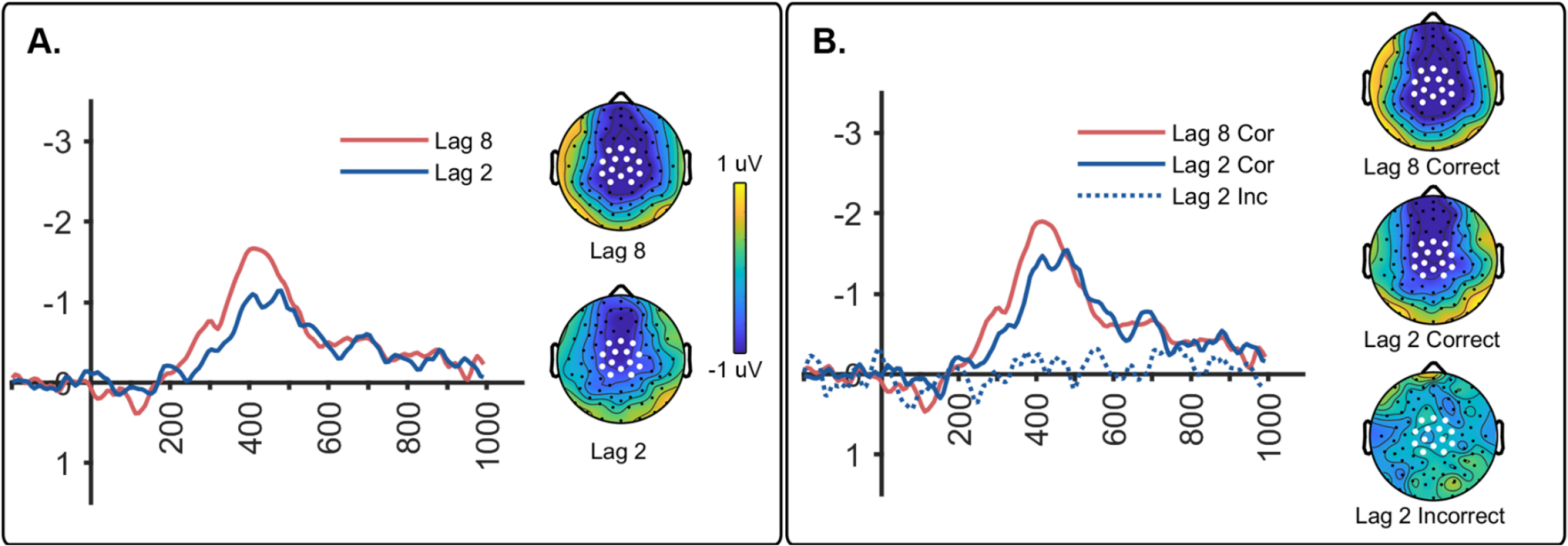
**(A)** The N400 waveforms (**left**) and topo maps (**right**) elicited by the test picture for lag 8 and lag 2 conditions combined across correct and incorrect trials. The sensors used for measuring mean amplitude are indicated by white dots. **(B)** The N400 waveforms (**left**) and topo maps (**right**) from Experiment 2 elicited by the test picture (T2) separated into correct and incorrect trials. Topo maps are averaged activity in a window from 300 to 500 ms

The N400 component produced by the test picture was computed as the difference between unrelated and related (*unrelated* – *related*) ERPs. Figure 4A shows N400 waveforms as well as topo maps collapsed across correct and incorrect trials. A paired *t*-test revealed that the N400 amplitude was significantly different for lag 8 relative to lag 2 (*t*(19) = 2.231, *p* = 0.038, *d* = 0.499). In addition, N400 amplitude was greater than zero for both lag 2 (*t*(19) = 5.047, *p* < 0.001, *d* = 1.129) and lag 8 (*t*(19) = 4.963, *p* < 0.001, d = 1.110).

N400 waveforms and topo maps separated by correct versus incorrect relatedness judgments are shown in Fig. 4B. Data for lag 8 are shown only for correct trials as there were too few trials in the incorrect lag 8 condition to create a meaningful ERP. The results suggest that the amplitude of the N400 was larger for the two correct conditions (lags 2 and 8) relative to the incorrect condition (lag 2 incorrect). This was evaluated with a repeated-measures ANOVA, which revealed a significant effect of condition (*F*(2, 38) = 8.354, *p* = 0.004, η^2^_*p*_ = 0.305). Bonferroni post hoc tests revealed that the N400 amplitude for the lag 2 incorrect condition was smaller than correct conditions at both lags (lag 2: *p* = 0.020 and lag 8, *p* < 0.001). The correct conditions for lags 2 and 8 were not significantly different (*p* > 0.05).

We also examined whether the N400 in each condition was significantly different from zero. One-sample *t*-tests against zero microvolts confirmed that both correct conditions were significantly different from zero (lag 2: *t*(19) = −4.303, *p* < 0.001, *d* = −0.962 and lag 8: *t*(19) = −5.381, *p* < 0.001, *d* = −1.000). In contrast, N400 amplitude for lag 2 incorrect did not differ from zero (*t*(19) = −0.474, *p* = 0.641, *d* = −0.106). In addition, we examined whether semantic priming was eliminated in the lag 2 incorrect condition by comparing it to zero microvolts using a Bayesian two-tailed, one-sample *t*-test. We found an inverse Bayes factor of 3.890, which provides “substantial” evidence in favor of the null hypothesis that semantic priming is absent when participants are incorrect in identifying the prime picture.

### Discussion

In Experiment 2, the T2 target was the *prime* picture rather than the *test* picture as it was in Experiment 1. In this case, we are asking whether a blinked scene picture can still activate semantically related visual information that will prime a test picture. Because the test picture appears for a long duration at the end of the sequence, we assume that errors in judging whether it is related to T2 arise from failures to identify T2, which is presented for a short duration, visually masked, and blinked at the short lag (lag 2). The results show that the amplitude of the N400 elicited by the test picture was suppressed when the prime picture (T2) was blinked, similar to what occurred in Experiment 1 where the test picture was blinked.

In addition, semantic priming produced by the prime picture was reduced when it appeared during the AB. This suppression was due to an absence of N400 activity on incorrect trials in which participants were not aware of the identity of the blinked prime picture. In contrast, on correct trials, the amount of priming was comparable at the two lags. Experiments 1 and 2 together suggest that semantic priming reflected in N400 amplitude only occurs when the participants are aware of both prime and test pictures. The strong role played by awareness of prime and test pictures in the N400 priming effect does not fit with the idea that semantic priming is mediated by automatic processes that run outside of awareness. We consider this issue together with various qualifications about our conclusions in more depth in the *General discussion*.

## Experiment 3

Experiments 1 and 2 showed that semantic priming, as measured by the amplitude of the N400, depends critically on whether participants are aware of the picture presented at lag 2 during the AB. Robust N400s were observed for “aware” trials but were absent on “unaware” trials. These results are consistent with the idea that semantic priming does not occur automatically during stage 1 processing but instead is associated with the limited-capacity processing occurring in stage 2. In Experiment 3, we asked whether priming based on identity rather than semantic relatedness might occur automatically in stage 1. Identifying a visual scene would seem to be a necessary precursor to semantic analysis and might occur earlier, perhaps even in stage 1.

**Fig. 5:**
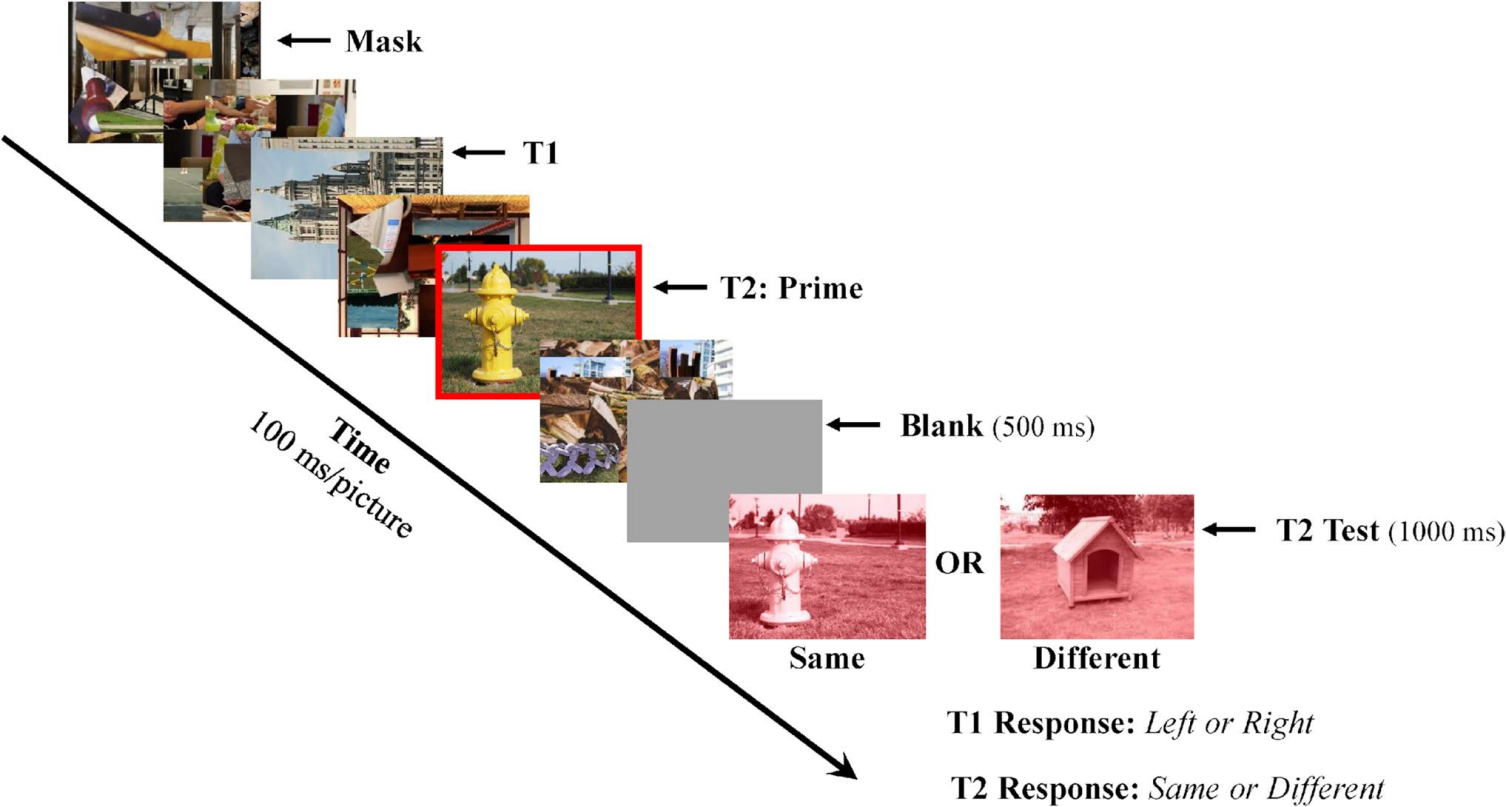
Schematic representation of a typical trial in Experiment 3. Picture streams contained incoherent background pictures (masks) and two targets (T1 and T2). T1 was a landscape/cityscape image that was rotated 90° to the left or right. T2 was the prime picture, and it was cued by a surrounding red frame. T2 occurred either two or eight pictures after T1. The stream ended with a final test picture (tinted red) that was either the same as or different from the prime picture (T2)

As in Experiment 2, the prime picture was presented at lag 2 or lag 8, relative to T1. The test picture was the final picture appearing in the stream and remained on-screen for 1,000 ms. It was either the same scene as the prime or a completely different, unrelated scene. The test pictures were converted to grayscale and tinted red to discourage the use of low-level color features of the scenes as a basis for inferring the identity of the prime and test pictures (see Fig. 5). Unlike Experiments 1 and 2, this procedure does not require participants to extract information about the relatedness of the test and prime pictures. If the identities of the prime and test pictures are extracted automatically in stage 1 processing, and if the generator of the N400 has access to these identities, we should observe identity priming even on trials in which the prime picture has been “blinked” and participants are mistaken in their identity judgments.

### Methods

#### Participants

Twenty-five individuals with normal or corrected-to-normal vision participated in the study (ages 18–28 years; 11 males, 14 females). To be included in the data set, participants needed at least 70% of their total EEG data to be usable. Five participants were excluded from analyses because they had too few usable segments as a result of excessive movements or noisy EEG recordings. The final sample size included 20 participants (ages 18–28 years; nine males; 11 females). On average, 58.5 trials (9.14%) were removed due to ocular artifacts and excessive noise in EEG recordings.

#### Design and procedure

The design of Experiment 3 closely resembled that of Experiment 2. The apparatus and stimuli were identical, as was the procedure for data collection and analysis. The same set of pictures was used with the following changes. The *related* test pictures from Experiment 2 were replaced by a repetition of the prime picture on *same* trials. The *unrelated* scenes from Experiment 2 were used as the *different* test pictures in Experiment 3. In addition, the test pictures were converted to grayscale and tinted red to discourage the use of low-level color features as a basis for matching (see Fig. 5).

The primary task for participants remained discrimination of T1 orientation, while the secondary task was now to report whether the test and prime pictures were the same or different. The prime picture (T2) was cued by a surrounding red frame. In addition, it was the only coherent scene in the stream other than T1. Subjects indicated their confidence in their identity decision using the same confidence rating scale employed in the previous experiments. Feedback was provided after each response.

### Results

#### Behavioral results

A correct trial was defined as accurate identification of T1 orientation (lag 2: *M* = 93.17%, *SE* = 0.62%; lag 8: *M* = 92.80%, *SE* = 0.65%), together with an accurate report of whether or not the test picture was the same as T2 (the prime picture). Confidence judgments had to be either *Sure* or *Not Sure* for the T2 response to be scored as correct; *Guess* responses were scored as incorrect. Behavioral results show that the average percentage accuracy averaged across *same* vs. *different* trials was significantly lower at lag 2 (*M* = 67.8%, *SE* = 2.5%) compared to lag 8 (*M* = 85.5%, *SE* = 7.2%), as revealed by a repeated-measures two-sample *t*-test (*t*(19) = 9.232, *p* < 0.001, *d* = 2.064). This indicates the presence of a strong AB effect, replicating behavioral results in Experiments 1 and 2.

#### ERP results

**Fig. 6:**
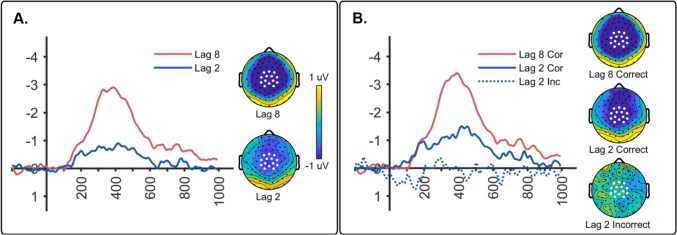
**(A)** The grand averaged N400 subtraction waveforms (*different* – *same*) waveforms and topo maps elicited by the test picture for lag 8 and lag 2 trials. Data are combined across correct and incorrect judgments of the identity of the T2 and test pictures. The sensors used for measuring mean amplitude are indicated by white dots. **(B)** The grand averaged N400 subtraction waveform (*different* – *same*) and averaged topo maps elicited by the test picture was between 300 and 500 ms, separated into trials in which identity judgments were correct or incorrect

The N400 elicited by T2 was isolated by subtracting “same” ERPs from “different” ERPs (*different* – *same*) and are shown in Fig. 6A for lags 2 and 8, collapsed across correct and incorrect trials. A single-sample *t*-test showed that the lag 8 N400 was larger than the lag 2 N400 (*t*(19) = 10.588, *p* < 0.001, *d* = 2.367). In addition, N400s were greater than zero for lag 8 (*t*(19) = 13.871, *p* < 0.001, *d* = 3.102) and lag 2 (*t*(19) = 5.283, *p* < 0.001, *d* = 1.181). These results replicate those of the first two experiments and show that even identity priming is reduced by the AB.

Figure 6B shows average N400s for correct and incorrect trials at lags 2 and 8. An omnibus repeated-measures ANOVA on N400 amplitude across conditions (lag 2 correct, lag 8 incorrect, and lag 8 correct) revealed a significant effect of conditions (*F*(2, 38) = 69.833, *p* < 0.001, η^2^_*p*_ = 0.786). Bonferroni post hoc tests revealed that the N400 for lag 2 incorrect was smaller than both correct N400s: lag 8 (*p* < 0.001) and lag 2 (*p* < 0.001). In addition, the N400 was larger for lag 8 correct compared to lag 2 correct (*p* < 0.001).

One-sample *t*-tests comparing N400 amplitude to zero microvolts were conducted to determine whether the N400 in each condition was greater than zero. N400 for both correct conditions met this criterion: lag 8 correct, *t*(19) = 13.791, *p* < 0.001, *d* = 3.084 and lag 2 correct, *t*(19) = 6.593, *p* < 0.001, *d* = 1.474. In contrast, the N400 for lag 2 incorrect was not significantly greater than zero (*t*(19) = 0.229, *p* = 0.822, *d* = 0.051). We followed these tests up with a two-tailed, Bayesian one-sample *t*-test against zero microvolts for the lag 2 incorrect condition and found an inverse Bayes factor of 4.203, which provides “substantial” evidence in favor of the null hypothesis that identity priming is absent when participants are incorrect in identifying the prime picture.

### Discussion

Experiment 3 examined whether repetition priming, as reflected in the N400 component, is affected by the AB. If identity priming, as measured by the N400 component, occurs in stage 1, it should be similar across lags even when conscious judgments of identity are severely impaired during the AB. Instead, we found that both behavioral accuracy and priming were reduced at lag 2 compared to lag 8. In addition, priming appeared to be absent on incorrect trials at lag 2, as shown by a “substantial” inverse Bayes factor in favor of the null hypothesis.

Overall, these results were similar to those of the first two experiments which examined *semantic* priming. In all three experiments, priming, as reflected in the N400 component of the ERP, was suppressed during the AB, and this suppression was generally associated with an absence of the N400 on incorrect trials. These results are consistent with the claim that *identification* of briefly presented visual scenes is impaired during the AB, presumably because, on some trials, the T2 scene picture (the prime) fails to gain access to stage 2 processing and awareness, which appears to be critical for correct identification of T2. Impairments in identification would also impair subsequent stages, such as semantic processing. This claim is supported by the finding that both repetition and semantic priming are similarly affected by the AB. For example, the amplitude of the N400 for correct responses at lag 2 was approximately the same for Experiments 2 (semantic priming) and 3 (repetition priming), which used similar paradigms (T2 was a prime picture followed by a clear test picture). Pesciarelli et al. (2007) reported similar findings when comparing semantic and repetition priming for words, and concluded that “semantic and repetition priming effects, under rapid serial visual presentation conditions, are modulated by at least partially overlapping neural mechanisms” (p. 21).

One exception to the claim of similar repetition and semantic priming effects is the finding in Experiment 3 that the N400 reflecting *repetition* priming on correct trials was much larger at lag 8 compared to lag 2 (Fig. 6B). The corresponding effect in Experiment 2 for *semantic* priming was not significant. This larger priming effect in Experiment 3 was unanticipated and we can only offer a conjecture as to its cause. One possibility is that conscious representations of the prime picture are generally clearer and/or more intact at lag 8 compared to lag 2 where attention is in short supply. This idea rests on the assumption that the clarity of conscious representations varies in a continuous rather than a discrete, all-or-none fashion, which seems to be supported by recent behavioral (Cohen et al., 2023) as well as ERP studies (Roth-Paysen et al., 2022). The clearer or more complete representations at lag 8 in Experiment 3 might elicit correspondingly larger N400s compared to less clear or more incomplete representations at lag 2 even when both representations lead to correct responses because perceptual matches should be “continuously” better with more complete representations. In contrast, the task in Experiment 2 relied on higher-level and more abstract representations (meaning), and it has generally been found that higher-level representations elicit a much smaller range of conscious representations compared to tasks using sensory or perceptual information (Jimenez et al., 2020, 2021; Windey & Cleeremans, 2015). In other words, compared to semantic priming, repetition priming is able to take advantage of the clearer or more complete representations that may be present at long lags in the AB paradigm.

It is also important to make a distinction between access to awareness and the nature of the representation in awareness. Previous authors (e.g., Cohen et al., 2023; Kouider et al., 2010) have suggested that *access* to awareness can be discrete at the same time that *what is being represented* in awareness is continuous. For example, Kouider et al., (2010, p. 306) pointed out that “Access consciousness can be graded in the sense that representations of an object can be more or less complete, whereas the mechanisms of access can still be all-or-none.” In Experiments 2 and 3, the magnitude of the blink was about the same, reflecting similar probabilities of T2 eliciting a conscious representation. However, the clarity or completeness of the resulting representations appeared to be different, particularly for lag 8 in Experiment 3.

## General discussion

Vogel et al., (1998, Experiment 2) reported a remarkable finding supporting the idea that the meaning of words can be activated outside of conscious awareness. They found that the ability to judge the semantic relatedness of two words (a prime followed by a target) was severely impaired when the target word was presented during the attentional blink. However, the N400 component of the ERP, which was used to measure semantic priming, was unaffected by the blink. This result is consistent with the idea that unlimited capacity processing in stage 1, which was thought to be restricted to relatively low-level visual features, actually includes processing of identity and meaning as well. However, conscious reporting of this information may require a second, higher-level processing stage that depends on access to attention (Zivony & Lamy, 2022), and it is this step that is impaired during the AB. We wondered whether this result, which was obtained with words, would also hold for analysis of visual scenes. On one hand, visual scenes are clearly more visually complex than words, and might therefore require access to higher-level processing. However, they are also the most common visual stimulus we encounter on a day-to-day basis, leading to rapid perception (Willems & Peelen, 2021), and potentially, automatic processing (Shiffrin & Schneider, 1977).

Across a series of three experiments, we used a variety of priming paradigms to examine whether the derivation of the identity and meaning of visual scenes is similar to the case of visually presented words in which case, semantic and repetition priming of scenes should be immune to the AB. In all three experiments, we found large AB effects for the ability to judge the semantic relatedness of two scenes, both when the *test* scene was blinked (Experiment 1) and when the *prime* scene was blinked (Experiments 2 and 3). However, unlike the results of Vogel et al., (1998, Experiment 2), semantic priming, reflected in the amplitude of the N400 component of the ERP, was also impaired by the attentional blink. The reduction in priming observed at the short lag during the blink was largely due to a complete lack of priming on those trials in which participants were unaware of the identity of the blinked picture and were therefore incorrect in reporting whether or not the two scenes were related. Similar results were found in Experiment 3 in which the prime and target scenes were identical (except for color) indicating that the AB also affected processing stages involved with identifying the scene.

In terms of bottleneck theories of the AB, these findings suggest that both identification and meaning of visual scenes depend on access to processing that occurs *after* the bottleneck that is responsible for the attentional blink (see also Zivony et al., 2018). This conclusion is consistent with a recent review by Zivony and Lamy (2022) on the mechanisms underlying AB that are reflected in ERP components. They concluded that the AB affects processes responsible for attentional engagement and encoding of information into working memory. In this view, failures in attentional engagement impair the extraction of meaning, resulting in decreases in the N400 component. The present findings are consistent with that conclusion.

Our results clearly differ from those reported by Vogel et al., (1998, Experiment 2), and it is tempting to conclude that differences between words and scenes are responsible for the conflicting results. However, there is also considerable variability in the pattern of results across those studies that used words as stimuli (see Zivony & Lamy, 2022). For example, Batterink et al. (2010) used a paradigm very similar to that of Vogel et al. (1998), except T1 was the printed name of a digit (“#SEVEN#”) rather than the string of identical digits (“7,777,777”) used by Vogel et al., (1998, Experiment 2). Batterink et al. (2010) found that the accuracy of relatedness judgments as well as the magnitude of semantic priming, as measured with the N400, were reduced at short lags in the AB. In addition, they separately examined correct and incorrect trials, and found a robust N400 for correct trials together with an absence of the N400 on incorrect trials. Both of these findings are similar to our results in Experiment 1, which used a similar paradigm with scenes instead of words.

Three subsequent studies used a method similar to that used in Experiment 2 of the present paper except lag was not varied. T2 always followed T1 at a short lag and was therefore subject to the AB. T2 was then followed at a long lag by T3, which was semantically related or unrelated to T2. The participant attempted to identify all three target words at the end of the trial. The N400 elicited by T3 provided a measure of the magnitude of priming from T2 on trials in which T2 was correctly or incorrectly reported. The question is whether T2 exerts equivalent priming effects on T3 regardless of whether T2 can be reported. Pesciarelli et al. (2007) examined both repetition and semantic priming during the blink. In agreement with the present results, they found that semantic and identity priming effects, as measured with the N400, were eliminated on trials in which participants were unable to report T2, i.e., on trials in which T2 was blinked. Similarly, Peressotti et al. (2012) found that T2 only produced priming effects when it was reported correctly. In contrast to these studies and the present results, Rolke et al. (2001) found significant N400 priming effects on prime-missed trials. In addition, they reported that the difference in the magnitude of the N400 priming effect for correctly versus incorrectly reported primes was only “marginally significant” (p < 0.08). The failure to find a significant reduction in the N400 priming effect on incorrect trials is consistent with the results of Vogel et al., (1998, Experiment 2), who found constant N400 amplitude across lags. However, the graphs in Rolke et al. (2001) show that the N400 was approximately three times larger on prime-recognized versus prime-missed trials, suggesting that the lack of a significant difference in this case was due to low power.

### Psychological refractory period and the attentional blink (AB)

Another approach to understanding conflicting findings in the AB is based on the psychological refractory period (PRP) paradigm. For example, Lien et al. (2008) sequentially presented participants with two stimuli (a tone followed by a printed word) with various temporal separations. The task was to make a speeded foot response to indicate the frequency of the tone and a speeded manual response to indicate whether the word was related or unrelated to a context word presented at the beginning of the trial. Responses to the word were delayed when it was presented at short lags (100–300 ms) relative to the tone, and because the stimuli occurred in different modalities and the responses used different effectors, Lien et al. suggested that this interference effect reflected competition for a *central resource* that is responsible for processes such as response selection. They also collected N400s to the words and they reasoned that if the N400 reflects automatic priming effects that occur independently of the conscious response, the amplitude and latency of this N400 should be unaffected by lag even when the behavioral response shows strong interference effects. Instead, they found that the amplitude of the N400 was reduced and its latency was increased at short temporal separations, indicating that the N400 priming measure and the speeded response to the word target displayed similar interference patterns. These results are similar to our Experiment 1 results using the AB.

In the Lien et al. (2008) study reviewed above, the tasks for T1 and T2 were different, and this was also the case for our studies and many of the other AB studies reviewed in this paper. For example, in our Experiment 1, participants judged the *orientation* of the T1 picture and the *semantic relatedness* between the T2 picture and a preceding context picture. This required them to adopt one task set for T1 and then quickly switch to a different task set for T2. At short lags, T2 may arrive before its task set has been activated, resulting in delayed responses or failures to process T2 altogether. Vachon and Jolicœur (2012) asked whether elimination of the task switch would eliminate the delay in processing of T2 and allow immediate high-level (semantic) processing of T2 at short lags. When task switching was required, they found results similar to those reported by Lien et al. (2008): the N400 to the second target was delayed and suppressed at short lags. However, these effects were eliminated in the no-switch condition. Responses to T2 as well as the N400 component elicited by T2 were no longer delayed or suppressed.

This raises the question of whether the presence or absence of task switching might explain the puzzling pattern of results in AB studies reviewed above. Perhaps studies in which there is no task switching between T1 and T2 also show no effect of the AB on amplitude and latency of the N400. Unfortunately, this does not appear to be the case. First, Vachon and Jolicœur (2012) found that the absence of task switching not only eliminated the delay in the N400, but also the delay in overt responding. In contrast, Experiment 2 of Vogel et al. (1998) reported a *dissociation* between overt responses and the latency and amplitude of N400 priming measures (e.g., Experiment 2 of Vogel et al., 1998). That is, overt responses showed substantial effect of the blink while the N400 priming measure was unaffected. Second, all of the AB studies reviewed in this paper involved task switching, and yet they produced very different results (e.g., Experiment 2 of Vogel et al., 1998, vs. Batterink et al., 2010).

### Load theory and the AB

Some researchers have attempted to account for these conflicting findings using “perceptual load theory” (Lavie & Tsal, 1994), which holds that there is a limited perceptual or attentional resource that can be shared between visual “objects” (letters, words, pictures, etc.). The amount of this resource that is allocated to any given object is determined by whether it is relevant or irrelevant as well as the difficulty of the particular task being carried out on that object. In the case of the AB, a difficult task being carried out on the task-relevant T1 will require a large investment of attentional resources resulting in a small allocation of attention to a T2 appearing at a short lag. This should result in a large blink compared to easy T1 tasks. Importantly, on trials in which the observer fails to identify T2, there will be less partial activation or priming of T2 as well (assuming that priming reflects activation of T2 that falls short of what is required for awareness). Therefore, load theory predicts that difficult T1 tasks will lead to larger blinks and less T2 priming compared to easy T1 tasks. One might then expect that results like those reported in Vogel et al. (1998) Experiment 2 will be observed whenever experimenters have chosen to use an easy T1 task, which should result in a small blink as well as substantial priming even on trials in which participants fail to identify T2.

These predictions regarding the effect of T1 task difficulty on priming during the AB were tested in a series of studies by Giesbrecht and colleagues (Giesbrecht et al., 2007; Sy et al., 2013). Giesbrecht et al. (2007) employed the same T2 task used in Experiment 2 of Vogel et al. (1998) in which participants had to judge the semantic relatedness of the T2 word to a previously presented context word. The amount of semantic priming was assessed using the amplitude of the N400 elicited by T2. In the *easy* T1 condition, they found that semantic priming, reflected in the N400 component, was unaffected by lag even though the accuracy of the relatedness judgments showed an AB, replicating the results of Experiment 2 in Vogel et al. (1998). Interestingly, in the *difficult* T1 condition, priming was eliminated during the blink. Both of these findings are in line with the predictions of load theory. A follow-up study by Sy et al. (2013) used the same stimuli as Giesbrecht et al. (2007), but found no significant difference in the N400 semantic priming (N400 amplitude) effect for a difficult versus easy T1 task. However, the difficulty manipulation did not affect the magnitude of the behavioral blink either, so these results are difficult to interpret.

Given the findings of Giesbrecht et al. (2007), can we conclude T1 task difficulty provides a useful principle for understanding when N400 priming effects will be independent of lag in the AB? That might be the case if we had some way to determine T1 task difficulty across the wide variety of paradigms used to study the AB. One possible approach would be to use the size of the AB as an index of T1 task difficulty based on the principle that difficult T1 tasks produce large AB effects (AB can be measured as the difference between the best and worst accuracy levels across lags; see Spalek et al., 2022, on measures of blink magnitude). Consider this approach applied to the conflicting results reported by Vogel et al. (1998) Experiment 2 versus Batterink et al. (2010). As pointed out earlier, Vogel et al. found robust N400 semantic priming effects that were unaffected by the AB. In contrast, Batterink et al. found that N400 amplitude was reduced during the blink and was absent on error trials, similar to the results of the studies reported here. This might suggest that Batterink et al. used a more difficult T1 task that used more of the available attentional resources, leaving less to be allocated to T2, resulting in a reduction of semantic priming during the blink. However, the *magnitude* of the blink was actually smaller in Batterink et al. (approximately 10%) compared to Vogel et al., Experiment 2 (approximately 25%), which suggests that, if anything, the T1 task used by Batterink et al. (2010) was *easier* than that used by Vogel et al. (1998), Experiment 2. One might conclude that blink magnitude is not an appropriate measure of perceptual load, but the inherent vagueness of load theory together with the lack of independent, reliable measures of perceptual load across a wide variety of tasks (Murphy et al., 2016) means that load theory is unlikely to shed light on the variability in results across studies examining the issue of whether semantic priming is independent of the blink.

### Effects of spatial attention

A different factor that might account for differences across studies in the effects of the AB on priming is spatial attention. The N400 appears to be strongly suppressed when words are presented in spatially unattended locations (McCarthy & Nobre, 1993; Vogel et al., 2005), which is consistent with the fact that spatial attention is known to suppress visual processing at early stages that precede higher-level processes such as semantic analysis (Hillyard & Picton, 1987). Studies in which the T1 task involves shifts of attention to a location that is different from the location of the T2 object would be expected to reduce the ability to identify T2 as well as eliminating its semantic priming effects because the identity of the T2 object would be suppressed at processing levels that precede semantic analysis. Is it possible that our finding that semantic priming is reduced by the AB is due to spatial attention effects? In the current study, the T1 task involved determining the orientation of scene pictures that were rotated 90° to the left or right. If this task involved shifts of attention to the tops of the trees or buildings in these pictures, then perhaps, at least some of the time, the critical object in T2 might appear in an unattended spatial location, resulting in an error in identifying T2 as well as eliminating its semantic priming effect. This seems unlikely, however, for the following reasons. Our T1 task did not involve discrimination of the shapes of the trees or buildings, which might depend on spatial attention. Instead, discrimination of the rotation direction can be accomplished using low frequency, global information in the scene because the tops of the scenes prior to rotation consist of a light region (corresponding to blue sky) while the bottom areas are dark (corresponding to the ground plane; see Baker et al., 2021, for an analysis of these scenes), so the T1 task involves determining whether the light band in the scene as a whole is on the left or right. In addition, this explanation would not apply to Experiment 3 because the T2 task was to determine if the prime and test pictures were “same” or “different,” and this can be accomplished without attending to the central object in the T2 scene. Importantly, the effects of the AB on semantic priming were similar to the preceding experiments.

Unfortunately, the reasons for the conflicting findings in this area remain unexplained. Perhaps the best we can do is to agree with Zivony and Lamy’s (2022) conclusion that “most studies show that the N400 amplitude is reduced during the AB, suggesting that semantic processing is often disrupted during the AB” (p. 407).

## Conclusions and limitations

There are conflicting findings regarding the question of whether visual stimuli such as words (Batterink et al., 2010; Giesbrecht et al., 2007; Rolke et al., 2001; Vogel et al., 1998) and objects (Harris & Little, 2010; Harris et al., 2010) are processed for meaning during the attentional blink. Several studies have indicated that semantic priming associated with words is unaffected by the blink (e.g., Shapiro et al., 1997a, 1997b; Vogel et al., 1998), suggesting that meaning is extracted during early perceptual processing stages that operate automatically and that the AB affects later stages involved in conscious report and working memory consolidation (Chun & Potter, 1995; see Dux & Marois, 2009, for a review).

In a series of three studies, we attempted to determine whether visual scenes presented during the AB are processed to a semantic level even when participants are incorrect in assessing the meaning of those scenes, as has been reported for the case of words (Luck et al., 1996; Experiment 2 in Vogel et al., 1998). This result might be expected given the ubiquity of scene processing in daily life as well as the importance of scene processing for survival. However, our results indicate that N400 measures of semantic priming of scenes are delayed (Experiment 1) and suppressed (Experiments 1 and 2) during the AB. This suppression effect is also observed for the case of repetition or identity priming (Experiment 3). These findings suggest that scene identification may occur during “late stages” of processing that are associated with conscious awareness, which runs counter to the idea that these operations are automatic and occur during early perceptual processing (e.g., Deacon et al., 2000; Kiefer, 2002; Vogel et al., 1998). A recent review of ERP components that are affected by the blink (Zivony & Lamy, 2022) reached a similar conclusion for words as well. However, it is important to note that, as Carl Sagan pointed out, “Absence of evidence is not evidence of absence” (Sagan, 1997). Our failure to find evidence of an N400 during the blink does not entail the conclusion that semantic processing of the blinked scene is absent. It is possible that meaning was extracted during the blink but this representation was not reflected in the N400 component. This could occur, for example, if the AB blocked the transmission of the information about meaning from earlier levels to higher-level processes that are involved in generating the N400. One might gain increased confidence in such a conclusion by using a wide variety of behavioral and neural measures but, in principle, we can never be sure about “accepting the null hypothesis.”

In addition, our conclusion that semantic analysis of scenes during the AB depends on awareness needs to be further qualified. Our task involved the analysis of the meaning of a central object in a scene. Scene perception also involves perception of other attributes such as category or gist of the scene (indoor, outdoor, beach, mountain, etc.). Slagter et al. (2010) showed that even these global properties of a scene show an AB. It would be interesting to determine whether these scene properties might show N400 priming effects that are invariant with lag in the AB.

## Data Availability

Data and materials (including stimuli) are available from the authors on written request.

## Code availability

Software (Python programs) used for data collection and analysis are available from the authors on written request.
