# Correction to: The N400 component reflecting semantic and repetition priming of visual scenes is suppressed during the attentional blink

**DOI:** 10.3758/s13414-024-03007-0

**Published:** 2025-01-17

**Authors:** Courtney Guida, Minwoo J. B. Kim, Olivia A. Stibolt, Alyssa Lompado, James E. Hoffman

**Affiliations:** 1 Department of Psychological and Brain Sciences, Boston University, Boston, MA USA; 2 Department of Biomedical Engineering, Ulsan National Institute of Science and Technology, Ulsan, Republic of Korea; 3 Department of Psychology, Florida International University, Miami, FL USA; 4 Department of Psychological and Brain Sciences, University of Delaware, Newark, DE 19716-2577 USA

**Correction to: Attention, Perception, & Psychophysics**

10.3758/s13414-024-02997-1

The funding source for co-author Minwoo Kim was inadvertently left out. The funding source information appears in two places in the article:

Current version

Author Note: This research was supported by an NSF EPSCoR grant 1632849 to the University of Delaware

New Version

Author Note: This research was supported by an NSF EPSCoR grant 1632849 to the University of Delaware and a grant from the National Research Foundation of Korea in support of Minwoo Kim: [Grant Number NRF-2020S1A3A2A02097375]

Current version

Funding NSF EPSCoR grant 1632849 to the University of Delaware

New Version

Funding NSF EPSCoR grant 1632849 to the University of Delaware and a grant from the National Research Foundation of Korea in support of Minwoo Kim: [Grant Number NRF-2020S1A3A2A02097375]

The original article has been corrected.

